# Transcriptome Dynamics during Maize Endosperm Development

**DOI:** 10.1371/journal.pone.0163814

**Published:** 2016-10-03

**Authors:** Jianzhou Qu, Chuang Ma, Jiaojiao Feng, Shutu Xu, Lei Wang, Feifei Li, Yibo Li, Renhe Zhang, Xinghua Zhang, Jiquan Xue, Dongwei Guo

**Affiliations:** 1 The Key Laboratory of Biology and Genetics Improvement of Maize in Arid Area of Northwest Region, Ministry of Agriculture, Northwest A&F University, Yangling, Shaanxi, China; 2 State Key Laboratory of Crop Stress Biology for Arid Areas, College of Life Sciences, Northwest A&F University, Yangling, Shaanxi, China; Huazhong University of Science and Technology, CHINA

## Abstract

The endosperm is a major organ of the seed that plays vital roles in determining seed weight and quality. However, genome-wide transcriptome patterns throughout maize endosperm development have not been comprehensively investigated to date. Accordingly, we performed a high-throughput RNA sequencing (RNA-seq) analysis of the maize endosperm transcriptome at 5, 10, 15 and 20 days after pollination (DAP). We found that more than 11,000 protein-coding genes underwent alternative splicing (AS) events during the four developmental stages studied. These genes were mainly involved in intracellular protein transport, signal transmission, cellular carbohydrate metabolism, cellular lipid metabolism, lipid biosynthesis, protein modification, histone modification, cellular amino acid metabolism, and DNA repair. Additionally, 7,633 genes, including 473 transcription factors (TFs), were differentially expressed among the four developmental stages. The differentially expressed TFs were from 50 families, including the bZIP, WRKY, GeBP and ARF families. Further analysis of the stage-specific TFs showed that binding, nucleus and ligand-dependent nuclear receptor activities might be important at 5 DAP, that immune responses, signalling, binding and lumen development are involved at 10 DAP, that protein metabolic processes and the cytoplasm might be important at 15 DAP, and that the responses to various stimuli are different at 20 DAP compared with the other developmental stages. This RNA-seq analysis provides novel, comprehensive insights into the transcriptome dynamics during early endosperm development in maize.

## Introduction

Maize (*Zea mays*) is one of the world’s most important crops and is used for food, animal feed and biofuel [1]. Development of the maize seed is initiated by double fertilization of a haploid egg cell and dikaryotic central cell to produce two filial structures: a diploid embryo and triploid endosperm. The endosperm occupies ~85% of the seed volume at maturity [2–4], providing nutrients and signals to the embryo throughout seed development and functioning as the site of starch and protein synthesis [5–6]. Thus, understanding the gene regulatory mechanisms involved in maize endosperm development is vital to improving seed yield and quality.

Development of maize endosperm is a very complex process that takes much longer than development of *Arabidopsis thaliana* endosperm [7–9]. Maize endosperm is histologically simple yet developmentally complex, with nuclear-type development; the primary endosperm nucleus undergoes several rounds of division without cytokinesis, forming a symplasm at 0~3 DAP in which many free nuclei occupy the peripheral cytoplasm surrounding a large central vacuole [2,8]. At 4~5 DAP, the endosperm cellularization process is essentially complete, and endosperm cell differentiation and proliferation begins. A phase of mitotic cell division that occurs after cellularization is largely responsible for generating the final population of endosperm cells. The endosperm grows rapidly, from 8 to 12 DAP, filling the entire seed cavity, and the maize endosperm cells gradually and asynchronously switch from a mitotic to an endoreduplication cell cycle. Cell division continues until approximately 20 to 25 DAP in the external cell layer of the endosperm, which develops into the aleurone and subaleurone layers. During the late period of endosperm development, programmed cell death occurs in the endosperm, as manifested by nuclear deformation and cell death following amyloid enrichment [2–5,7–8,10–11]. Studies have revealed that many key physiological progresses and most vigorous metabolic activities related to endosperm development usually occur before 25 DAP. Valuable information obtained from further analyses of gene activities during the early developmental stages (before 25 DAP) will allow for a deeper understanding of the developmental mechanisms of maize endosperm.

AS results in the generation of multiple mRNA transcripts from a single gene, which increases transcriptome complexity in plants in response to physiological and environmental changes [12–14]. Furthermore, AS is a central mode of genetic regulation that controls the developmental progression of maize endosperm [15–16]. At present, AS events have been identified and documented in several tissues of maize, including the ear, embryo, endosperm, leaf, root, shoot and tassel [14,17–18]. However, most previous studies have focused on only one developmental stage, and little attention has been paid to the dynamic AS events occurring in the endosperm at different developmental stages.

High-throughput next-generation sequencing (NGS) technologies are robust tools used in transcriptome analyses that enable increased understanding of the transcriptional regulatory mechanisms involved in maize endosperm development [6,14,19–21]. However, the NGS-based transcriptome study of endosperm development is still far from complete. For instance, gene expression patterns have not been extensively analysed at 5, 10, 15 or 20 DAP [6,14,20,22–23]. In addition, most previous analyses of AS events have focused on a single time point, ignoring the dynamics of endosperm development [14,16]. Finally, the functions of differentially expressed transcription factors (DETFs) during endosperm development need to be examined [6,14].

In this study, to further understand the genome-wide transcriptional regulation of endosperm development in maize, we applied next-generation high-throughput RNA-seq to perform transcriptomic analysis of maize endosperm at 5, 10, 15 and 20 DAP and explored the dynamic AS events and gene expression patterns during maize endosperm development. We also identified stage-specific AS events, genes and TFs that may be involved in developmental pathways. Our results enabled generation of a map of the transcriptional network and revealed the dynamic patterns of AS events and TFs associated with endosperm development in maize.

## Materials and Methods

### Ethics statement

The study was approved by the Ethics Committee of Northwest A&F University, Shaanxi, China. The experimental land was provided by Northwest A&F University and this study was carried out in strict accordance with the all relevant regulations. In the process of experiment, the study did not involve endangered or protected species and all necessary permits were obtained for the described study.

### Plant materials and conditions

The maize (*Zea mays*. *L*) hybrid Shandan 609, derived from the hybridization of chang 7–2 (male) and 91227 (female), was grown under field conditions at approximately 67,500 plants ha-1 in the summer of 2013 in Yangling, Shaanxi Province, China. The ears were bagged before silk emergence, followed by manual self-pollination. Three biological replicates of the ears were harvested at 5, 10, 15 and 20 days after self-pollination and then the endosperm tissues of the different biological replicates were separated by removing all tissues, from the pedicle up to the hilar region, after cutting open the pericarp along the edge of the hilar region using sterilised forceps and a surgical blade under a stereomicroscope (S1 Fig). To ensure for the integrity and specificity of the endosperm tissues and the absence of contamination by any other tissues, all slightly damaged endosperm tissues were discarded. Then, 60, 40, 15 and 15 complete endosperm tissues corresponding to 5, 10, 15 and 20 DAP were placed in three 2.0 mL RNase-free tubes and frozen immediately in liquid nitrogen. The tubes containing the samples were stored at -80°C prior to RNA extraction.

### RNA isolation, library construction and transcriptome sequencing

Total RNA of endosperm from three biological replicates was extracted separately from each sample using TRIzol-A+ Reagent (Invitrogen) following the manufacturer’s instructions. The concentration of purified RNA was quantified using a Q5000 spectrophotometer (Quawell, San Jose, CA, USA), and RNA integrity was evaluated with an Agilent 2100 Bioanalyzer (Agilent Technologies, Santa Clara, CA, USA). An amount of 10μg RNA per sample was used for RNA sequencing. Maize endosperm mRNA sequencing libraries were constructed according to the standard Illumina protocol. RNA enrichment was achieved with an NEBNext Poly(A) mRNA Magnetic Isolation Module (NEB, E7490). RNA-seq libraries were prepared using an NEBNext mRNA Library Prep Master Mix Set for Illumina (NEB, E6110) and NEBNext Multiplex Oligos for Illumina (NEB, E7500). Fragments were size-selected by 1.8% agarose gel electrophoresis, followed by PCR amplification with a Library Quantification Kit-Illumina GA Universal (Kapa, KK4824). All established RNA-seq libraries were sequenced to generate 2×100-nucleotide paired-end reads with the Illumina HiSeq^TM^ 2000 platform.

### Read cleaning and mapping

Clean RNA-seq reads from each sample were obtained using Trimmomatic software (http://www.usadellab.org/cms/?page=trimmomatic), which filters raw reads containing segments with low sequencing quality scores (quality score < 20) and/or containing two or more ambiguous nucleotides (Ns). The quality of the clean reads was examined using FASTQC software (http://www.bioinformatics.babraham.ac.uk/projects/fastqc/). The clean reads were aligned to the maize B73 reference genome (ZmB73_RefGen_v2; http://www.maizesequence.org/) with Tophat2 (v2.09; http://ccb.jhu.edu/software/tophat/index.shtml) program using Bowtie2 read alignment software [24,25], allowing for two mismatches and one insertion and/or deletion (indel).

### Detection of AS events

AS events supported by the RNA-seq data were detected and visualized using the SpliceGrapher software (v0.2.2; http://splicegrapher.sourceforge.net), which makes meaningful predictions even for genes with low read coverage, discriminates between real and spurious splice sites, and can improve the reliability of detection of AS [26]. Six major types of AS events were recognized: exon skipping (ES), intron retention (IR), alternative 3' splicing (A3SS), alternative 5' splicing (A5SS), alternative first exon (AFE) splicing and alternative last exon (ALE) splicing. To further verify the newly identified transcribed regions, the reads that mapped to the reference gene model were visualized using Integrative Genomics Viewer (IGV) software (v2.3.34; https://www.broadinstitute.org/igv/) [27]. Additionally, all alternative splice sequences of the genes were extracted and used to identify protein domains according to the Pfam database (v29.0; http://pfam.xfam.org/) [28].

### Estimation of gene expression abundance

The transcripts of the maize hybrid Shandan 609 were first assembled from the clean reads using Cufflinks software (v2.1.0; http://cole-trapnell-lab.github.io/cufflinks), and they were then used to update the annotated maize gene models (http://www.maizesequence.org) using Cuffmerge program (http://cole-trapnell-lab.github.io/cufflinks/cuffmerge). Transcripts with lengths of shorter than 200 bp were further filtered. The normalised expression level of each gene was estimated by calculating fragments per kilobase of transcript per million mapped reads (FPKM) values with Cufflinks software. The FPKM method eliminates the influences of genetic differences in length and sequence and can be used to compare gene expression between samples [29]. DEGs were identified by comparing two biological conditions using EBseq software [30], which estimates the variance of RNA-seq data without biological replicates. This program provides posterior probabilities (P-values) by adjusting for multiplicity using the Benjamini-Hochberg procedure [31], and utilizes corrected P-values to determine false discovery rates (FDRs). Detection of DEGs was based on an FDR was <0.01 and no less than a two-fold change (log_2_ ratio value of >1 or < −1) in FPKM between the two conditions.

### Statistical analysis

For a given gene set, enrichment analysis of Gene Ontology (GO) terms was performed using the online tool AgriGO (http://bioinfo.cau.edu.cn/agriGO/analysis.php) [32]. The significance levels (*p*-values) of the GO terms were determined with Fisher’s exact test and adjusted with the Benjamini-Hochberg algorithm for multiple comparisons. A GO term was considered significantly enriched if the adjusted *p*-value was lower than 0.05. REVIGO (http://revigo.irb.hr/) was further used to summarize and visualize the enriched GO term sets in the non-redundant mode [33]. Gene expression patterns were identified by the K-mean clustering analysis with the squared Euclidean distance measure, this analysis was performed based on 15 clusters determined using the Calinski-Harabasz (CH) index [34]. Further, hierarchical clustering analysis was performed with the R package pheatmap (https://cran.r-project.org/web/packages/pheatmap/index.html) using Pearson’s correlation coefficient as the distance measure.

### Real-time quantitative PCR

To verify the gene expression levels and AS events from determined by RNA-seq, quantitative real-time PCR was performed using SYBR green I (Bio-Rad) and a CFX96 Real-time PCR detection system (Bio-Rad, Hercules, USA). Three biological replicates were tested for each time point. Total RNA (10 μg per sample) was reverse transcribed using a FastQuant RT Kit (with gDNase) (TIANGEN, Beijing, CN) following the manufacturer’s instructions (10 μL of reaction mix, including 2 μL of 10×Fast RT Buffer, 1 μL of RT Enzyme Mix, 2 μL of FQ-RT Primer Mix and 5 μL of RNase-Free ddH_2_O). Gene-specific primers were designed using the Primer Premier 5.0 software (Premier, CAN). To identify homologous sequences and to ensure for primer specificity, the target-specific gene sequences and primer pair sequences were blasted against the non-redundant (Nr) database and Primer-BLAST (GenBank, NCBI), respectively. The secondary structures of the gene sequences as well as the dimers and hairpin structures of the primer pair sequences, were analysed to further ensure for primer specificity. All primers were synthesised by Shenggong Corporation (Shenggong, CN). The gene-specific primer pair sequences are listed in S1 Table. Quantitative real-time PCR was performed to determine transcript abundances in a total volume of 20 μL (10 μL of 2×SuperReal PreMix Plus (SYBR Green) (TIANGEN, Beijing, CN), 0.6 μL of forward primer, 0.6 μL of reverse primer, 4.8 μL of RNase-free ddH_2_O and 4 μL of template). The thermal cycling conditions were as follows: 95°C for 15 min and 40 cycles of 95°C for 10 s, 60°C for 20 s and 72°C for 30 s, followed by a melting curve programme (95°C for 10 s, followed by an increase from 65 C° to 95 C° in 5 s increments of 0.5°C). Real-time PCR was performed for validation of alternatively spliced gene expression, then the products were resolved on a 1.5% agarose gel and stained with ethidium bromide to confirm the generation of specific products with the correct sizes. The housekeeping gene Actin (gene ID: *GRMZM2G082484*) was used as an endogenous reference and an internal control to normalise the C_T_ values of the target genes in the same run [35]. The normalised C_T_ values were manually filtered using a cut off of 35. The relative expression levels were calculated as previously described [36]. The C_T_ values of the target genes are listed in S2 Table.

## Results

### Transcriptome sequencing of endosperm at four developmental stages

To obtain an overview of the transcriptional profile during early endosperm development in maize, we utilized the Illumina HiSeq2000 platform to perform paired-end RNA-seq of the endosperm tissues at 5, 10, 15 and 20 DAP. After removing low-quality sequencing reads (reads with a quality score of smaller than 20 and/or reads containing two more ambiguous bases (Ns)), we obtained 49,371,842, 48,483,950, 50,254,726 and 44,555,238 clean reads from the endosperm tissues at 5, 10, 15 and 20 DAP, respectively (Table 1). We further mapped these clean reads to maize reference genome sequences (ZmB73_RefGen_v2; http://www.maizesequence.org) with Tophat2 (v2.09) software [24], allowing for two mismatches and one indel. The proportion of mapped reads aligned to the B73 genome sequences (70.66% ~ 75.26%) was comparable to those reported in other maize transcriptome studies [37]. The proportion of uniquely mapped reads was greater than 95% (Table 1). Furthermore, coverage of the reads mapped to the maize genome sequences varied across the four stages (S2 Fig), indicating that the transcriptome is dynamic during the early stage of maize endosperm development.

**Table 1:** Statistical results for sequenced and mapped reads to the maize B73 genome.

Stage	Clean reads	Mapped reads (%)	Unique mapped (%)	Multiple mapped (%)
**5 DAP**	49,371,842	75.18	95.95	4.05
**10 DAP**	48,483,950	70.66	95.27	4.73
**15 DAP**	50,254,726	75.26	96.02	3.98
**20 DAP**	44,555,238	74.92	95.29	4.71

### Dynamics of alternative splicing during maize endosperm developmental stages

**Fig 1:**
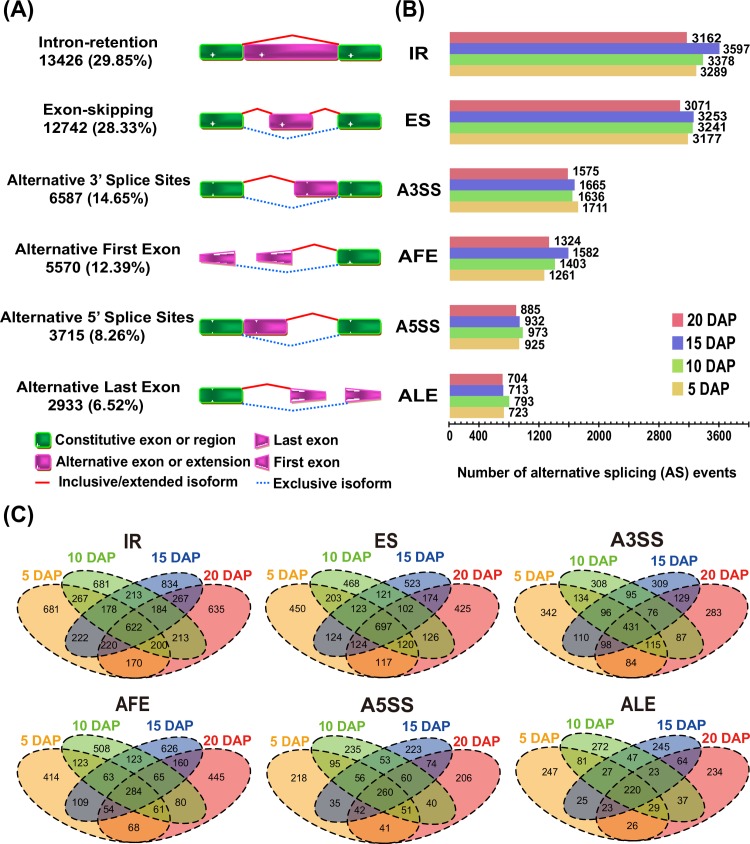
Overview and identification of AS events during endosperm development. (A) Alternative splicing (AS) events were categorized into the six most frequent types: alternative 3’ splicing (A3SS), alternative 5’ splicing (A5SS), alternative first exon (AFE) splicing, alternative last exon (ALE) splicing, intron retention (IR) and exon skipping (ES). The total number of all types of AS events and their frequencies are shown; these numbers include newly identified and annotated AS events. (B) The bar chart presents the number of AS events detected at the four developmental stages. (C)The Venn diagrams depict shared and unique AS events among the four developmental stages of maize endosperm.

Using SpliceGrapher (v0.2.2) [26], we identified 44,973 AS events for 11,010 protein-coding genes at four developmental stages (S3 Table). These AS events could be grouped into the following six categories: A3SS (6,587, 14.65%), A5SS (3,715, 8.26%), AFE (5,570, 12.39%), ALE (2933, 6.52%), IR (13,426, 29.85%) and ES (12,742, 28.33%) (Fig 1A). Among these categories, IR was the most prevalent AS event observed during maize endosperm development. This finding is in agreement with a previous study of embryo and endosperm tissues of early developing seeds at 9 DAP [14]. ES was also found to be a predominant AS event in our study (Fig 1A). This result is in contrast with previous studies of *A*. *thaliana*, which have reported low levels of ES (<5%) [38,39].

**Fig 2:**
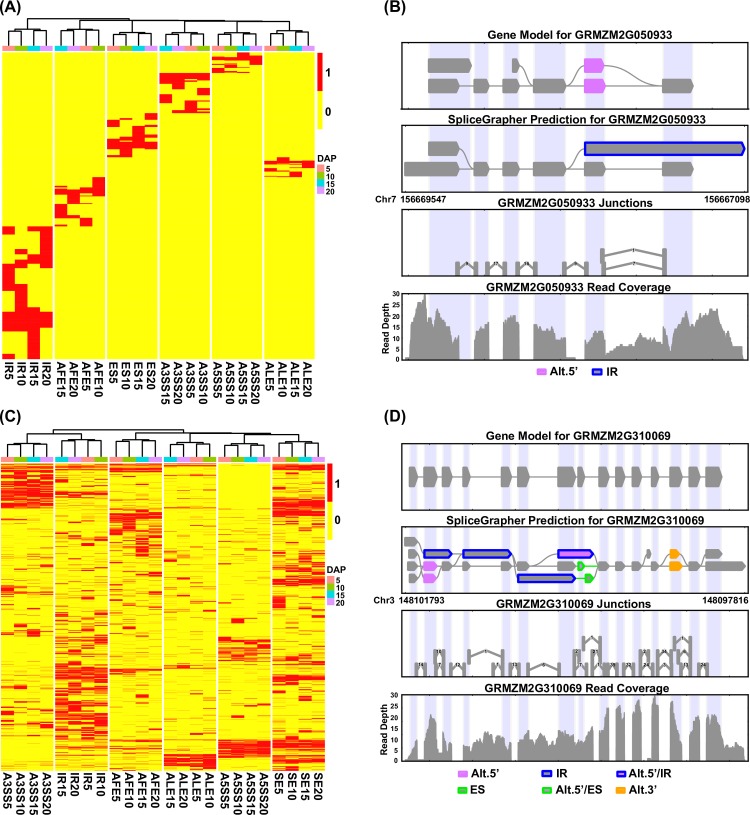
Discovery of a single AS event or multiple AS events in maize endosperm genes. (A) The heat map shows the dynamic changes in 5,905 genes with a single AS event during endosperm development, and the scale bar indicates whether an event occurred (red: event occurrence; yellow: no event occurrence). (B) An example of a single AS event occurring in a newly identified transcribed region (chromosome 7: 129960922–129965733); this developmentally regulated splicing event was only observed at 15 DAP. (C) Dynamic variation in multiple AS events in 5,116 genes is indicated at several developmental stages (the scale bar is defined in A). (D) An example of multiple AS events in *GRMZM2G051866*, indicating the diversity of newly identified transcribed regions (chromosome 10: 130393296–130404103) and the various developmentally regulated splicing events that occur at this locus during different developmental stages.

Further analysis revealed that dynamic AS events occurred during maize endosperm development at 5, 10, 15 and 20 DAP (Figs 1B–1C and 2). We found that the numbers of the six types of AS events differed among the four developmental stages (Fig 1B). For instance, 3,162, 3,597, 3,378 and 3,289 IR events occurred at 5, 10, 15 and 20 DAP, respectively (Fig 1B). Furthermore, a large number of maize genes exhibited stage-specific AS patterns (Fig 1C). For instance, 5,905 genes only exhibited one of the six types of AS events during one developmental stage (Fig 2A and S4 Table). These genes were belonged to several TF families, including the *CAMTA* (*GRMZM2G447551*), *Dof* (*GRMZM2G456452* and *GRMZM2G176063*), *GATA* (*GRMZM2G163200* and *GRMZM2G104390*), *GRF* (*GRMZM2G096709*), *WOX* (*GRMZM2G031882* and *GRMZM2G409881*), and *YABBY* (*GRMZM2G088309* and *GRMZM2G529859*) families, as well as the *bHLH* family. *GRMZM2G050933* is an example of a newly identified transcribed gene with IR event (chromosome 7: 156667822–156668205). The mapped reads further verified this transcribed region (S3A Fig), and its developmentally regulated splicing event was only observed at 5 DAP (Fig 2B). We also extracted all of the exon sequences, including the retained intron sequence, and identified a novel protein domain (cyclin, C-terminal domain) using the Pfam database (S1 Table). *GRMZM2G412075* is an example of a gene exhibiting the second most frequently observed type of AS event; this gene contained a novel junction, and this developmentally regulated splicing event was only observed at 15 DAP (S4 Fig). We next extracted the complete exon sequences, in addition to all of the complete, non-overlapping exon sequences, and Pfam analysis showed that the protein domain belonged to the RRM_1 and poly-adenylate binding protein (PABP) families. Interestingly, the position of the protein domain formation (Poly-adenylate binding protein, PABP) moved forward to the 5’ end in sequence and the length of annotation decreased when the overlapping exon sequences were removed (S1 Table). We also detected genes with A5SS or A3SS events (S5 and S6 Figs), and some genes with AS events were selected for validation by qRT-PCR (S7 Fig). Rigorous GO analysis revealed the functional enrichment of genes with single AS events in diverse biological processes, such as intracellular protein transport, signal transmission, cellular carbohydrate metabolism, cellular lipid metabolism, lipid biosynthesis, and protein modification (S5 Table and S8A Fig). Additionally, a comparable number of maize genes (5,116) showed multiple AS events during the four developmental stages (Fig 2C and S6 Table). A typical example is *GRMZM2G310069*, a gene encoding a hypothetical protein that may regulate secondary metabolism (the protein domain is Amidohydro_3). This gene not only presented with multiple AS events at different stages but also provided a preliminary map of temporal AS events (Fig 2D and S3B Fig). Genes with multiple AS events were also significantly enriched in many key biological processes, including histone modification, tRNA processing, cellular amino acid metabolism, DNA repair and intracellular transport (S7 Table and S8B Fig). These results indicate that AS is a vital event that generates proteins with diverse functions to regulate endosperm development in maize.

### Dynamic changes in maize endosperm gene expression at four developmental stages

The sequenced reads in this study were valuable for investigating gene expression in maize endosperm during the four developmental stages. By assembling transcripts with clean reads using Cufflinks software (v2.1.0) [29], the boundaries of 19,164 annotated maize genes could be further extended, and 2,504 putative new genes were found to be expressed at these four stages (S8 Table). Significant BLAST hits for a proportion of these putative new genes were obtained in the Nr (96.34%), SwissProt (52.08%), GO (70.13%), KEGG (16.25%) and COG (14.85%) databases (S9 Table). Among these genes, 59 appear to have roles in transcriptional regulation (S9 Table).

**Fig 3:**
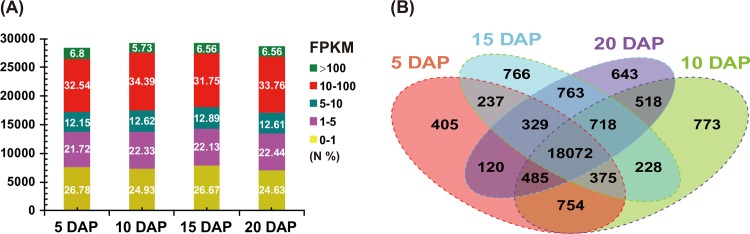
Analysis of global gene expression based on FPKM values among different endosperm developmental stages. (A) Distribution of the transcripts at five expression levels based on the FPKM values are shown, and the expression values from 10 to 100 correspond with the most highly represented transcripts at the four developmental stages. The vertical axis represents the number of transcripts; and ‘N’ is the percentage of transcripts expressed at the corresponding level. (B) A total of 25,186 genes (FPKM value ≥ 1) were expressed during the four stages. Genes with shared and specific expression were detected during these stages.

To gain a global overview of gene expression during the four examined developmental stages, gene expression abundance was estimated by calculating FPKM values (Fig 3A and S10 Table). Less than 7% of the genes were highly expressed (FPKM > 100), whereas more than 30% had FPKM values ranging from 10–100. An FPKM value of ≥ 1 was observed for 20,777, 21,923, 21,488 and 21,648 genes expressed at 5, 10, 15 and 20 DAP, respectively, and 18,072 genes were consistently expressed at all four developmental stages (Fig 3B). Further, 14703 (50.3%) and 19714 (68.6%) expressed genes reported by Chen *et al*. (2014) overlapped with our data at 10 and 20 DAP, respectively. However, 19714 (72.8%) expressed genes reported by Li *et al*. (2014) overlapped with our data at 10 DAP. These differences may have resulted from the use of different maize varieties. In our study, we used the maize hybrid Shandan 609, while Chen *et al*. (2014) and Li *et al*. (2014) examined the maize inbred line B73, and used two different methods to filter gene sets, which likely led to the differences in the numbers of expressed genes. We expected that our data would be enriched for the maize endosperm transcriptome. Furthermore, 405, 773, 766 and 643 genes with diverse biological processes, cellular components, and molecular function annotations were specifically expressed at 5, 10, 15 and 20 DAP (Fig 3B), respectively. For instance, genes specifically expressed at 5 DAP were mainly involved in transferase activity and the transferring of hexosyl groups (S11 Table). In contrast, significant enrichment for transcriptional regulation, the carbohydrate catabolic process, and the defence response was observed among the genes specifically expressed at 10 DAP (S11 Table). These results are in agreement with earlier reports showing that the active accumulation of storage compounds begins at approximately 10 DAP [6,20]. Additionally, the genes specifically expressed at 15 DAP were mainly involved in lipid and protein metabolism (S11 Table), and those specifically expressed at 20 DAP were closely related to transcriptional regulation and carbohydrate and lipid metabolic processes (S11 Table).

**Fig 4:**
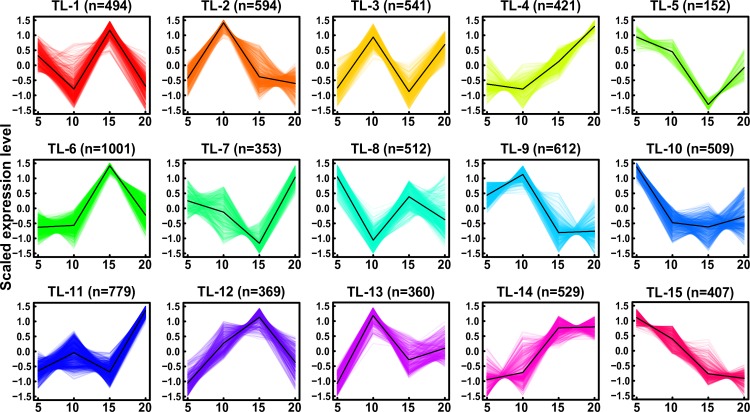
Expression patterns of DEGs in developing maize endosperm. Fifteen expression patterns were characterized by the fluctuating expression of gene sets at 5, 10, 15 and 20 DAP. The DEG sets showed different tends of transition from down- to up-regulated states or from up- to down-regulated states over the developmental stages (TL-14 and 15). The up- and down-regulated gene sets are staggered or are depicted consecutively over the developmental stages of maize endosperm. The scaled expression levels of the DEGs are provided on the *y-*axis, the developmental stages are shown on the *x*-axis, the coloured lines represent the individual gene expression clusters, and the trend in expression of each gene set is depicted as a black line. ‘n’ represents the number of DEGs.

**Fig 5:**
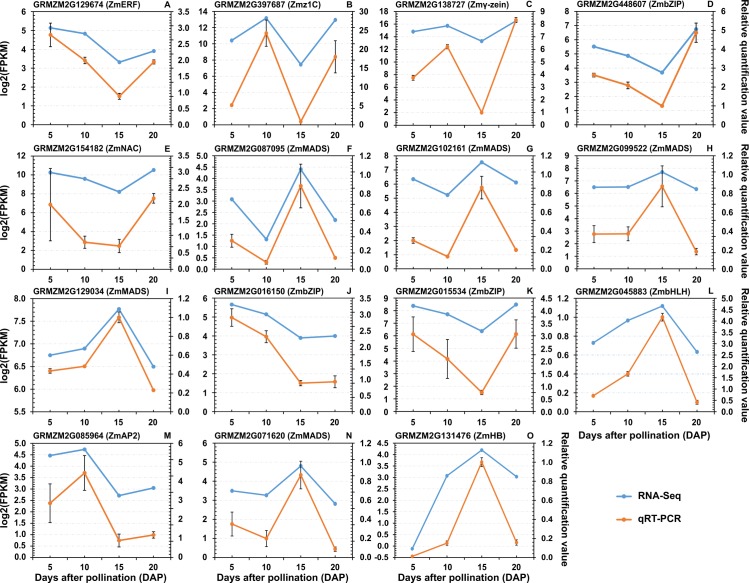
Expression pattern analysis of 13 TF and 2 zein genes by qRT-PCR and RNA-seq. The *y-* axis shows the mRNA levels. The scale on the right indicates gene expression level based on RNA-Seq. The scale on the left shows relative gene expression levels based on qRT-PCR. The *x*-axis indicates the day of endosperm sampling after pollination. The letters correspond to the genes. The blue lines correspond with RNA-seq; and the gold lines correspond with qRT-PCR.

To further identify dynamic changes in gene expression during early maize endosperm development, we carried out hierarchical clustering analysis of genes that were differentially expressed among the different development stages. Genes with no less than a two-fold change (log_2_ ratio value of >1 or < −1) and FDR of ≤ 0.01 were considered differentially expressed. We identified 7,633 DEGs among the different developmental stages using EBSeq [30] (Table 2 and S12 Table), and then grouped them into fifteen clusters (TL-1 –TL-15) based on the CH index [34] (Fig 4 and S13 Table). The DEGs in TL-2, TL-5, TL-6, TL-7, TL-9, TL-12 and TL-13 showed a single transition point at 10 or 15 DAP. In contrast, the DEGs in TL-1, TL-3, TL-8, and TL-11 exhibited two transition points at both 10 and 15 DAP. qRT-PCR analysis of 15 genes, including 13 TF and 2 zein genes, was performed to further confirm the dynamics of differential gene expression (Fig 5 and S2 Table). The qRT-PCR results revealed similar expression trends compared with the RNA-seq results. Additionally, the DEGs in the A, D, E, H, I, J, K, L and O clusters identified in qRT-PCR showed a single transition point at 15 DAP, whereas those in the B, C, F, G, M and N clusters showed two transition points at both 10 and 15 DAP. (Fig 5). These results facilitate the identification of co-expressed gene sets associated with regulatory nodes that are involved in the temporal control of maize endosperm development.

**Table 2:** Differentially expressed genes among four endosperm developmental stages in maize.

Comparison	DEG number	Up-regulated	Down-regulated	No.
5 DAP vs 10 DAP	2803	1679 (59.9%)	1124 (40.1%)	D10_5
5 DAP vs 15 DAP	3402	1956 (57.5%)	1446 (42.5%)	D15_5
5 DAP vs 20 DAP	3490	2255 (64.6%)	1235 (35.4%)	D20_5
10 DAP vs 15 DAP	3566	1676 (47.0%)	1890 (53.0%)	D15_10
10 DAP vs 20 DAP	2938	1454 (49.5%)	1484 (50.5%)	D20_10
15 DAP vs 20 DAP	2802	1716 (61.2%)	1086 (38.8%)	D20_15

**Fig 6:**
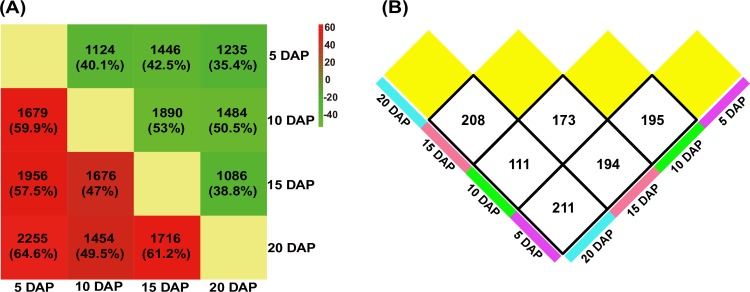
Distributions of the numbers of DEGs and DETFs. (A) Identification of the dynamic distribution of DEGs (fold change (FC) ≥ 2 (for up-regulation) or FC ≤ 0.5 (for down-regulation)) in developing endosperm via comparison of two stages; the red colour represents up-regulation, and green represents down-regulation. The scale bar depicts the percentages of DEGs in both directions (up and down). (B) A semi-matrix showing the dynamic changes in the DETFs, as determined by pair-wise comparisons of their expression at different developmental stages.

Stringent GO enrichment analysis of the DEGs in these clusters revealed that those in TL-2, TL-6, TL-7, TL-10, TL-13 and TL-15 predominantly participated in the response to monocarboxylic acid metabolic process, defense response and lipid metabolic process, glucan biosynthetic process and cellular carbohydrate biosynthesis, translation, oligopeptide transport, and cellular nitrogen compound metabolic process, respectively (Table 3 and S14 Table). Additionally, some DEGs exhibited similar expression patterns and thus may be involved in the same biological process, for example, a fraction of the TL-1 and TL-8 DEGs exhibited a down-up-down expression pattern at three transition points and were enriched for translation and nucleosome assembly (Table 3 and S14 Table). As the previous study, histone family genes have been shown to be crucial for the packaging of DNA and cell cycle regulation [40]. Compared with other clusters, the DEGs in TL-3, TL-4, TL-5, TL-9, TL-11.TL-12 and TL-14 were not significantly enriched in a specific GO biological process, while they were significantly enriched in diverse molecular functions: TL-4 (three-step: down-up-up) was enriched in 2 iron, 2 sulphur cluster binding; TL-5 (three-step: down-down-up) was enriched in macromolecular complex; TL-9 (three-step: up-down-down) was enriched in catalytic activity; TL-12 (three-step: up-up-down) was enriched in monooxygenase activity; TL-14 (three-step: up-up-up) was enriched in serine-type endopeptidase activity; while TL-3 and TL-11 (three-step: up-down-up) was especially enriched in nutrient reservoir activity, which occurs primarily in the starchy endosperm [6] (S14 Table). The largest proportion of up-regulated genes was identified in the comparison between 5 and 20 DAP, whereas the largest proportion of down-regulated genes was detected in the comparisons between 10 and 15 DAP and between 10 and 20 DAP (Fig 6A). These results indicate that the gene expression patterns change dramatically during maize endosperm development from 5–20 DAP.

**Table 3:** Functional category enrichment analysis of genes in fifteen major clusters.

Cluster	GO_acc	GO Term	Query	Background	p-value	FDR
			Item/total	Item/total		
**TL-1**	GO:0006412	translation	77/324	1071/25,288	2.50E-34	1.80E-31
	GO:0034645	cellular macromolecule biosynthesis	113/324	3777/25,288	9.50E-19	2.40E-16
	GO:0006334	nucleosome assembly	20/324	206/25,288	1.20E-11	7.70E-10
**TL-2**	GO:0032787	monocarboxylic acid metabolic process	11/324	188/25,288	4.30E-05	2.60E-02
**TL-3**	GO:0045735	nutrient reservoir activity	10/313	118/25,288	4.10E-06	1.30E-03
**TL-4**	GO:0051537	2 iron, 2 sulfur cluster binding	5/136	40/25,288	4.20E-06	7.80E-04
**TL-5**	GO:0032991	macromolecular complex	13/74	2352/25,288	1.90E-02	1.00E+00
**TL-6**	GO:0006952	defense response	10/534	82/25,288	2.10E-05	1.50E-02
	GO:0006629	lipid metabolic process	32/534	674/25,288	3.30E-05	1.50E-02
**TL-7**	GO:0009250	glucan biosynthetic process	8/196	88/25,288	7.90E-07	2.00E-04
	GO:0034637	cellular carbohydrate biosynthesis	10/196	168/25,288	1.30E-06	2.00E-04
**TL-8**	GO:0006412	translation	78/326	1071/25,288	6.00E-35	4.30E-32
	GO:0006333	chromatin assembly or disassembly	27/326	244/25,288	1.70E-16	3.20E-14
	GO:0006334	nucleosome assembly	25/326	206/25,288	3.20E-16	3.20E-14
**TL-9**	GO:0003824	catalytic activity	122/205	11249/25,288	1.20E-05	3.30E-03
**TL-10**	GO:0006412	translation	27/268	1071/25,288	3.90E-05	1.40E-02
**TL-11**	GO:0045735	nutrient reservoir activity	12/407	118/25,288	1.10E-06	5.20E-04
**TL-12**	GO:0004497	monooxygenase activity	11/213	429/25,288	1.30E-03	4.20E-01
**TL-13**	GO:0006857	oligopeptide transport	5/210	65/25,288	2.80E-04	9.00E-02
**TL-14**	GO:0004252	serine-type endopeptidase activity	7/182	213/25,288	1.10E-03	3.90E-01
**TL-15**	GO:0034641	cellular nitrogen compound metabolic	11/139	373/25,288	9.00E-06	3.30E-03

### Dynamic expression of TFs during maize endosperm development

A total of 1,945 maize TFs were found to be expressed in at least one of the four developmental stages (S15 Table); of them, 473 TFs were differentially expressed (S16 Table). Furthermore, more than 100 TFs were found to be differentially expressed in each of the six comparisons (Fig 6B). The most highly differentially expressed genes were members of the *bZIP* family. A representative example is *OPAQUE2* (O2; *GRMZM2G015534*), a *bZIP* TF with FPKM values of 333.47, 210.00, 82.90 and 357.73 at 5, 10, 15 and 20 DAP, respectively. The expression pattern of O2 was consistent with that observed in the qRT-PCR validation experiments. O2 plays an important role in the regulation of seed carbon and nitrogen metabolism and activates α- and *β-prolamin* genes in the synthesis of storage proteins during endosperm development [41–44].

**Fig 7:**
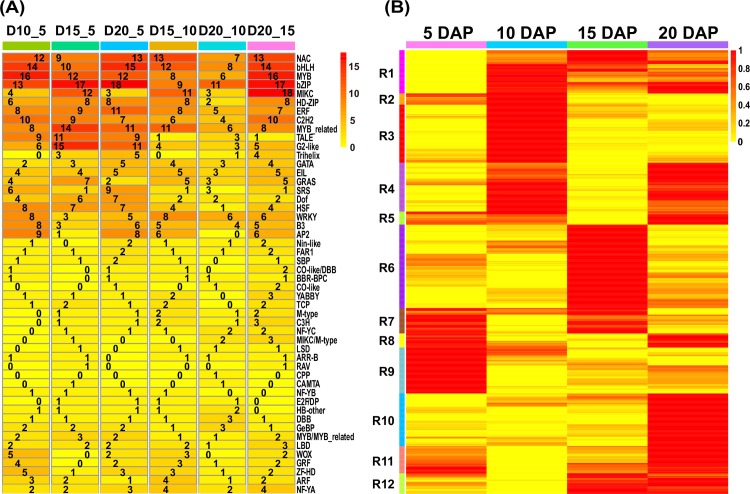
Enrichment and expression analysis of DETFs during maize endosperm development. (A) Pair-wise comparisons identified the dynamic distribution of the numbers of DETFs from 50 TF families. The scale bar represents the number of DETFs. (B) Heat map of DETFs identified at the time point of their peak expression based on normalised FPKM values. Twelve modules are classified according to normalised FPKM values of more than 0.5. The scale bar shows the normalised FPKM values.

The DETFs were distributed among 50 TF families; of them, 40% (20/50) contained DETFs in each cluster (Fig 7A). In contrast, 60% (30/50) of the TF families included relatively fewer DETFs, with most exhibiting either up- or down-regulation at specific stages of endosperm development (Fig 7A).

The dynamic expression of DETFs was demonstrated by hierarchical clustering analysis (Fig 7B). A total of 473 DETFs could be grouped into 12 clusters; of these DETFs, 39 (R2, R5, R7-9 and R11), 44 (R1-5), 40 (R1, R6-7 and R12) and 44 (R1, R4-5, R8 and R10-12) were highly expressed specifically at 5, 10, 15 and 20 DAP, respectively (Fig 7B and S17 Table). GO enrichment analysis showed that the DETFs that were highly expressed at 5 DAP were mainly involved in binding processes, such as water, ice, zinc ion, protein, cation, ion, metal ion and transition metal ion binding, as well as nucleus and ligand-dependent nuclear receptor activity (S18 Table). Specifically, TFs in the *C3H*, *FAR1*, *GATA*, *GeBP* and *ZF-HD* families are key regulators of the above biological processes. The DETFs at 10 DAP were involved in various immune responses, signalling, binding and lumen development, as well as other biological functions (S18 Table). In particular, *Dof*, *E2F/DP*, *GATA*, *GRF*, *SRS*, *WOX* and *ZF-HD* family members have important roles in regulating the above functions, and the functions of some of these TFs have been reported in previous studies [14,45–46]. In addition, the *GRF* members differentially expressed at 10 DAP are mainly involved in the regulation of nucleotide binding in the endosperm. To a certain extent, many biological processes overlapped at 15 and 10 DAP, but the different DETFs at 15 DAP showed significant enrichment for protein metabolic processes and cytoplasmic localisation. These biological processes were found to involve several DETF families, such as *CAMTA*, *Dof*, *GeBP*, *MIKC/M-type*, *M-type*, *SRS* and *WOX* (S18 Table), and DETFs exhibiting high expression in the R6 cluster were also identified at specific stages. Although the biological functions regulated by the DETFs at 20 DAP were similar to those regulated at the other stages, many of the DETFs at 20 DAP were involved in the responses to various stimuli, binding, and formation of the lumen and were components of organelles, the membrane, and the nucleoplasm in contrast with those at 15 DAP (S18 Table). DETFs in the *BBR-BPC*, *CO-like*, *E2F/DP* and *ZF-HD* families showed the greatest contribution to these biological processes, whereas those in R10 were specifically observed at 20 DAP.

Overall, these results indicate that members of these 50 TF families either play important roles in regulating the entire developmental process or act as regulatory nodes to control endosperm development at specific times.

## Discussion

In the present study, we performed high-throughput transcriptome sequencing to study the transcriptome dynamics during maize endosperm development from 5 to 20 DAP. These findings further enrich the existing research literature.

First, analysis of the RNA-seq data resulted in the identification of 44,973 AS events in 11,010 protein-coding genes. ES was found to be a predominant AS event in maize endosperm development (Fig 1A), in contrast with the reported findings of a low level of ES (<5%) in *Arabidopsis thaliana* [38–39]. Further, unlike previous studies of AS events [12–18], dynamic AS events were observed in the present study (Figs 1C and 2). These AS events were not only stage-specific but also underwent dynamic changes, and the biological diversity of gene function was affected by single and multiple AS events. As exemplified in *GRMZM2G050933* and *GRMZM2G310069*, to a certain extent, AS events resulted in the addition or removal of protein domains, as well as the rearrangement of sequences, allowing for the comprehensive examination of the biological diversity of gene function. GO enrichment analysis revealed that although the biological functions of genes with single or multiple AS events differed, these genes participated in developmentally related pathways (S5 and S7 Tables). Thus, the AS data represent a valuable resource for identification of candidate genes involved in endosperm development, especially because approximately 40% of maize genes have not yet been functionally annotated in the Ensembl Plant database (http://plants.ensembl.org/). A representative candidate is the uncharacterized gene *GRMZM2G310069*, which exhibited a different AS type in each of the four developmental stages (Fig 2D). The above results and previous studies suggest that AS plays an important role in the transition between developmental stages during endosperm development.

Second, differential expression analysis indicated that the gene expression programmes changed dramatically during maize endosperm development between 5 and 20 DAP. A total of 7,633 DEGs in fifteen clusters were identified through pair-wise comparisons between each of the four developmental stages (Fig 4). DEGs in 73.33% (11/15) of the clusters exhibited transition points at 10 and/or 15 DAP, and these points were confirmed by qRT-PCR (Fig 5 and S2 Table). These findings demonstrate the importance of these two time points during maize endosperm development. This observation is supported by the results of previous studies showing that at approximately 10 DAP, endosperm cells asynchronously and gradually switch to an endoreduplication cell cycle, with notable accumulation of spherosomes and protein bodies, and the cytoplasm becomes dense, rapidly filling the seed cavity, resulting in a change in cell shape [7,11]. Several important developmental changes, such as storage protein and starch accumulation and an abrupt increase in the IAA concentration, begin at approximately 10 DAP [47]. In addition, many significant metabolic changes, including changes in cyclin proteins activity, starch accumulation and GA signalling pathway activity, occur at 15 DAP [11,22,48].

A detailed investigation of the expression patterns of the DEGs was performed to elucidate the differences in biological functions within and among of 15 AS gene sets. In contrast with previous studies [6,49], trend analysis performed here allowed for determination of the expression trends of various functional gene sets, including zein genes, which account for 70% of corn endosperm proteins and affect the translucency of the mature kernel [50,51]. In addition, “up-and-down” oscillating expression patterns were identified in zein gene subfamilies. Almost all of the DEGs among *19-kD α-zein*, *22-kD α-zein*, *15-kD β-zein*, *16-kD*, *27-kD γ-zein* and *50-kD γ-zein* exhibited “up-down-up” oscillating expression patterns in the TL-2, -3, -9, -11 and -13 clusters; these findings are consistent with those of a previous study [49]. These oscillating expression patterns were also confirmed by qRT-PCR for *22-kD α-zein (GRMZM2G397687)* and 27-*kD γ-zein (GRMZM2G138727)* (Fig 5 and S2 Table). Nevertheless, two *19-kD α-zein* genes (*GRMZM2G053120* and *GRMZM2G404459*) in TL-10 and an *18-kD δ-zein* (*GRMZM2G100018*) gene in TL-7 exhibited “down-up-down” and “down-down-up” oscillating expression patterns. The oscillating expression patterns of these zein genes are likely related to their positional expression in maize endosperm. For example, *19-kD α-zein* and *22-kD α-zein* are expressed in a narrow vertical strip on the adgerminal side of the endosperm at 10 DAP. By 15 DAP, *22-kD α-zeins transcripts* are detectable in most regions; conversely, *10-kD σ-zeins transcripts* are only observed on the adgerminal and abgerminal sides of the endosperm, and *19-kD B1α-zein transcripts* are enriched in peripheral, central and starchy endosperm cells. At 20 DAP, *19-kD B1α-zein* transcripts are present in most regions, *22-kD α-zein transcripts* are enriched in the peripheral regions of the endosperm, and *15-kD β-zein transcripts* are enriched in the border region of the abgerminal side of the endosperm [52]. Obviously, further investigation of the expression patterns of DEGs is necessary to delineate the mechanisms that control gene expression programmes during maize endosperm development.

Finally, the differential regulation of TFs occurred during endosperm development. A total of 473 DETFs from 50 families were identified during the four developmental stages of maize endosperm (Fig 6B and S16 Table), most of which have been identified to be involved in the regulation of plant development (S9 Fig). A representative example, a *bZIP* TF termed OPAQUE2 (O2; *GRMZM2G015534*), was expressed at a high level and exhibited a consistent expression pattern between RNA-seq and qRT-PCR results in our study (Fig 5 and S2 Table). Previous reports have also shown that O2 mainly participates in the regulation of carbon and nitrogen metabolism and activates the α- and β-prolamin genes in the synthesis of storage proteins during endosperm development [41–44]. Furthermore, the present study revealed that *WRKY* family members were involved mainly in the physiological programmes of senescence, pathogen defence and phytohormone signalling. These results are in agreement with previous reports showing that *WRKY* family members play roles in plant immunity and plant senescence, as well as salicylic acid- and abscisic acid-mediated plant defence and abiotic stress tolerance [53,54]. Although the differentially expressed members of the *ARR-B*, *GeBP* and *ARF* families were expressed at relatively low levels, these TFs are involved in hormone signalling, including cytokinin signalling (*ARR-B* and *GeBP*) and auxin signalling (*ARF*). Moreover, some of the changes in the biological functions of these DETF family members were specific to only one of the four stages of endosperm development (Fig 7A). Fig 7B shows the results of complete analysis of the DETF expression patterns, with the highly expressed DETFs depicted according to the specific developmental stages. Further functional analysis revealed the potential regulatory roles of several DETFs in the transition between developmental stages (S18 Table). These findings may facilitate the generation of a stage-specific regulatory network and delineation of the regulatory mechanisms involved in maize endosperm development.

## Supporting Information

S1 Fig Typical morphologies of maize kernel and endosperm at four days after pollination (DAP). (TIF)

S2 Fig Read coverage for the four time points studied. A, B, C and D show reads mapped to maize reference genome sequences at 5, 10, 15 and 20 DAP, respectively. The boxes in red depict the differing coverage of the reads mapped to the maize genome sequences across the four stages.
(TIF)

S3 Fig Reads mapped to the reference gene model. A, an example illustrating reads mapped to the gene (*GRMZM2G050933*) model, with a single AS event observed at 5 DAP. B, an example showing reads mapped to a gene (*GRMZM2G310069*) model, with multiple AS events observed at 10 DAP. The different colours represent different bases, and the coloured areas indicate the regions in which AS events occurred. Corresponding to Fig 2B and 2D.
(TIF)

S4 Fig Exon skipping (ES) identification. A and B, an example illustrating a newly identified transcribed regions, *GRMZM2G412075*, showing reads mapped to the gene model, with a single AS event observed at 15 DAP. The different colours represent different bases, and the coloured areas indicate the regions in which ES occurred.
(TIF)

S5 Fig Alternative 5’ splice site (Alt.5’) identification. A and B, an example illustrating newly identified transcribed regions of *GRMZM2G036619*, showing reads mapped to the gene model, with a single AS event observed at 5 DAP. The different colours represent different bases, and the coloured areas are regions with an A5SS.
(TIF)

S6 Fig Alternative 3’ splice site (A3SS) identification. A and B, an example illustrating newly identified transcribed regions of *GRMZM2G053588*, showing reads mapped to the gene model, with a single AS event observed at 5 DAP. The different colours represent different bases, and the coloured areas are regions with an A3SS.
(TIF)

S7 Fig Validation of alternative splicing events by RT-PCR. The capital letters indicate types of AS events. Corresponding to S1 Table.
(TIF)

S8 Fig Gene Ontology (GO) enrichment analysis of genes with AS events in maize endosperm. A, Enrichment of biological process-related GO terms of genes with a single AS event. B, Enrichment of biological process-related GO terms of genes with multiple AS events.
(TIF)

S9 Fig Gene Ontology (GO) enrichment analysis of differentially expressed transcription factors (DETFs). Enrichment of biological process-related GO terms of DETFs from four developmental stages of maize endosperm.
(TIF)

S1 Table Primer pair sequences and domain information for genes with an AS event. (XLS)

S2 Table qRT-PCR analysis of 15 genes. (XLS)

S3 Table Alternative splicing (AS) types of genes. (XLS)

S4 Table Single alternative splicing (AS) events in genes. (XLS)

S5 Table GO annotation of genes with a single alternative splicing (AS) event. (XLS)

S6 Table Multiple alternative splicing (AS) events in genes. (XLS)

S7 Table GO annotation of genes with multiple alternative splicing (AS) events. (XLS)

S8 Table Cufflinks analysis of putative genes. (XLS)

S9 Table Functional annotation of putative genes. (XLS)

S10 Table Gene expression at four developmental stages. (XLS)

S11 Table GO annotation of genes differentially expressed at specific stages. (XLS)

S12 Table Differentially expressed genes. (XLS)

S13 Table Differentially expressed gene clustering. (XLS)

S14 Table GO annotation of differentially expressed gene clusters. (XLS)

S15 Table Transcription factor (TF) expression at four developmental stages. (XLS)

S16 Table Differentially expressed transcription factors (DETFs). (XLS)

S17 Table Clustering of differentially expressed transcription factors (DETFs). (XLS)

S18 Table GO annotation of differentially expressed transcription factors (DETFs) at a specific stage. (XLS)
