# Supplementary figures and images for: Transcriptome Dynamics during Maize Endosperm Development
Source: PLoS One. 2016 Oct 3;11(10):e0163814. doi: 10.1371/journal.pone.0163814 (PMC5047526; doi:10.1371/journal.pone.0163814)

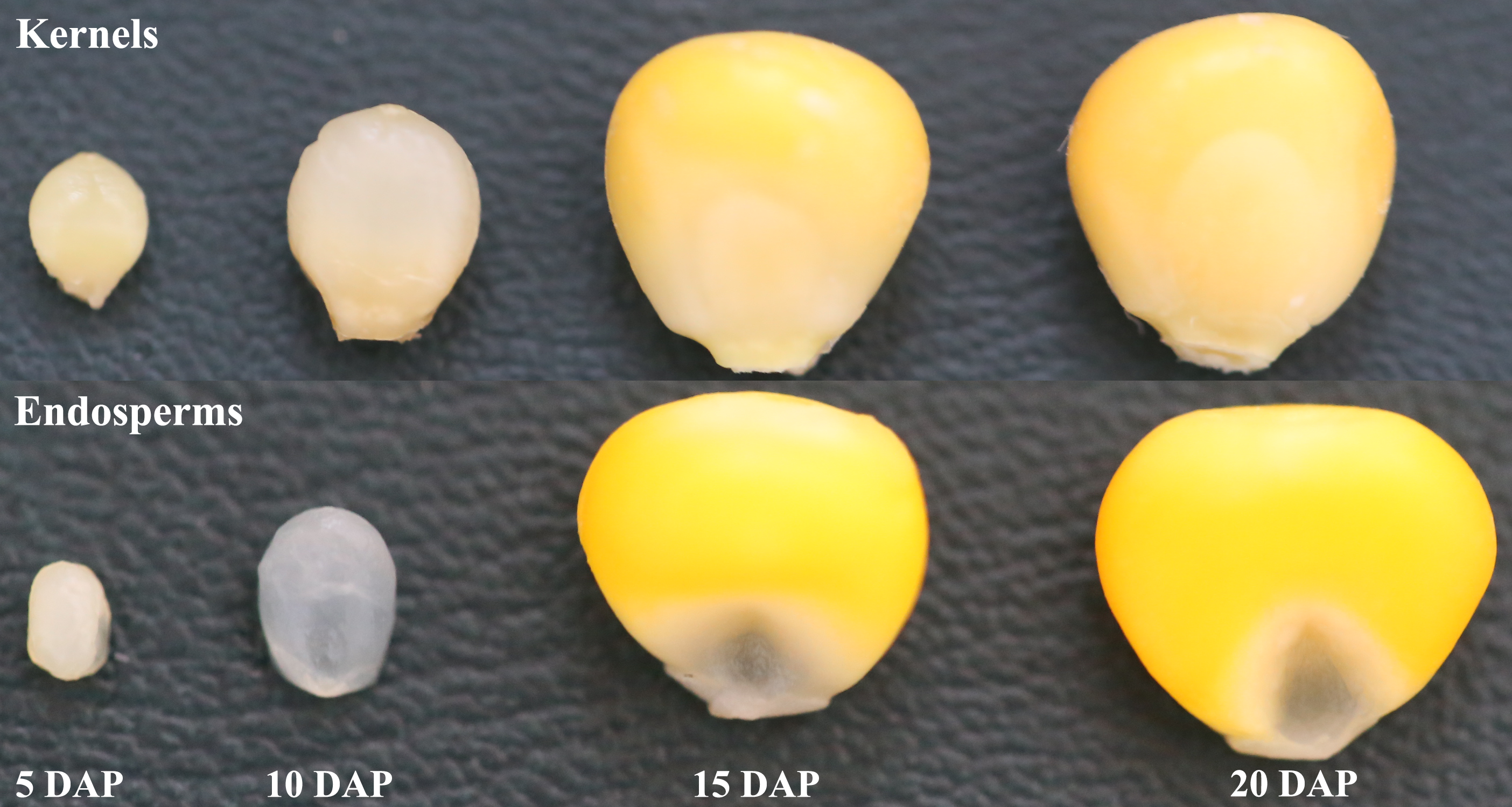

Supplement: S1 Fig — (TIF) [file pone.0163814.s001.tif]

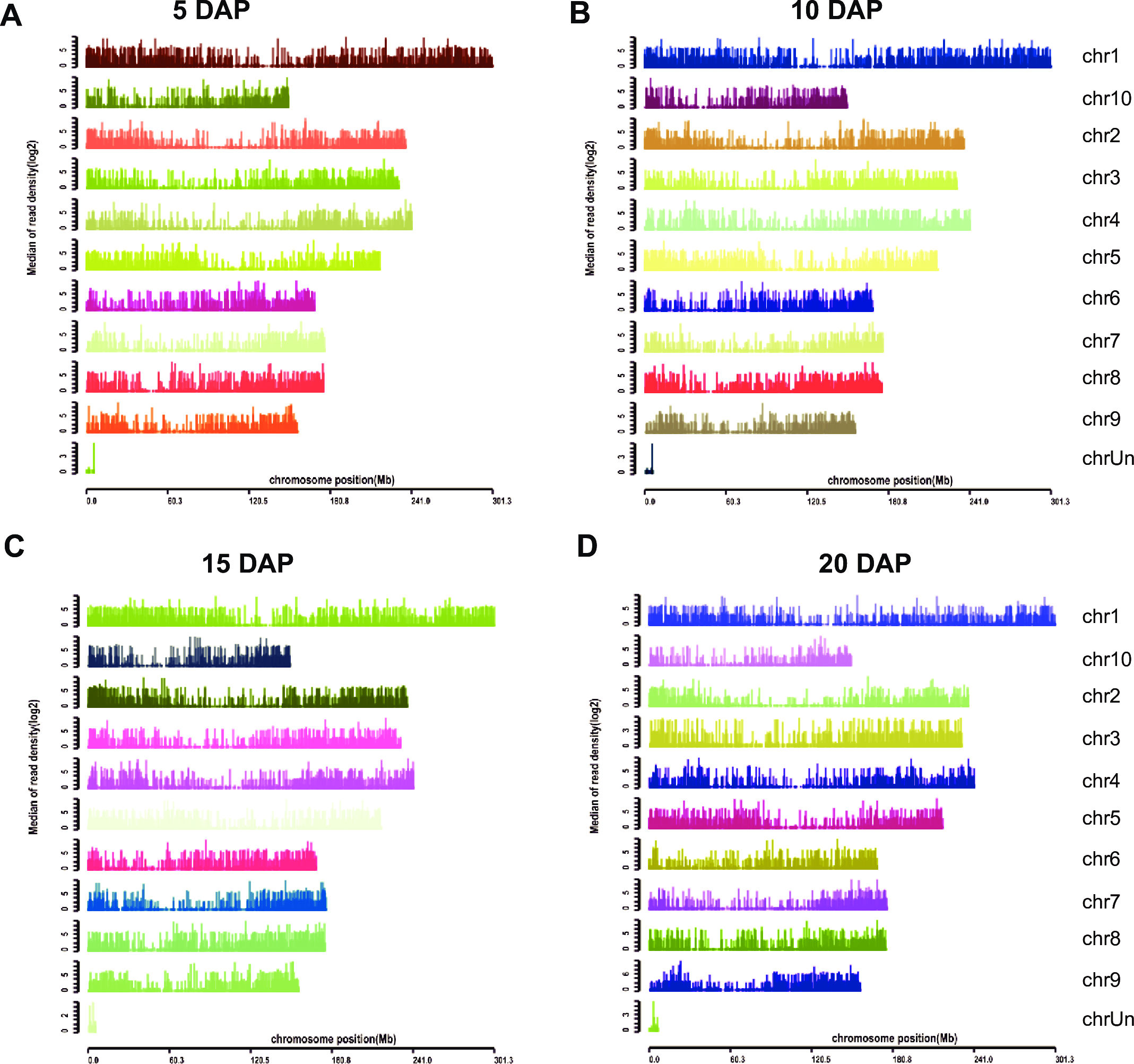

Supplement: S2 Fig — A, B, C and D show reads mapped to maize reference genome sequences at 5, 10, 15 and 20 DAP, respectively. The boxes in red depict the differing coverage of the reads mapped to the maize genome sequences across the four stages. (TIF) [file pone.0163814.s002.tif]

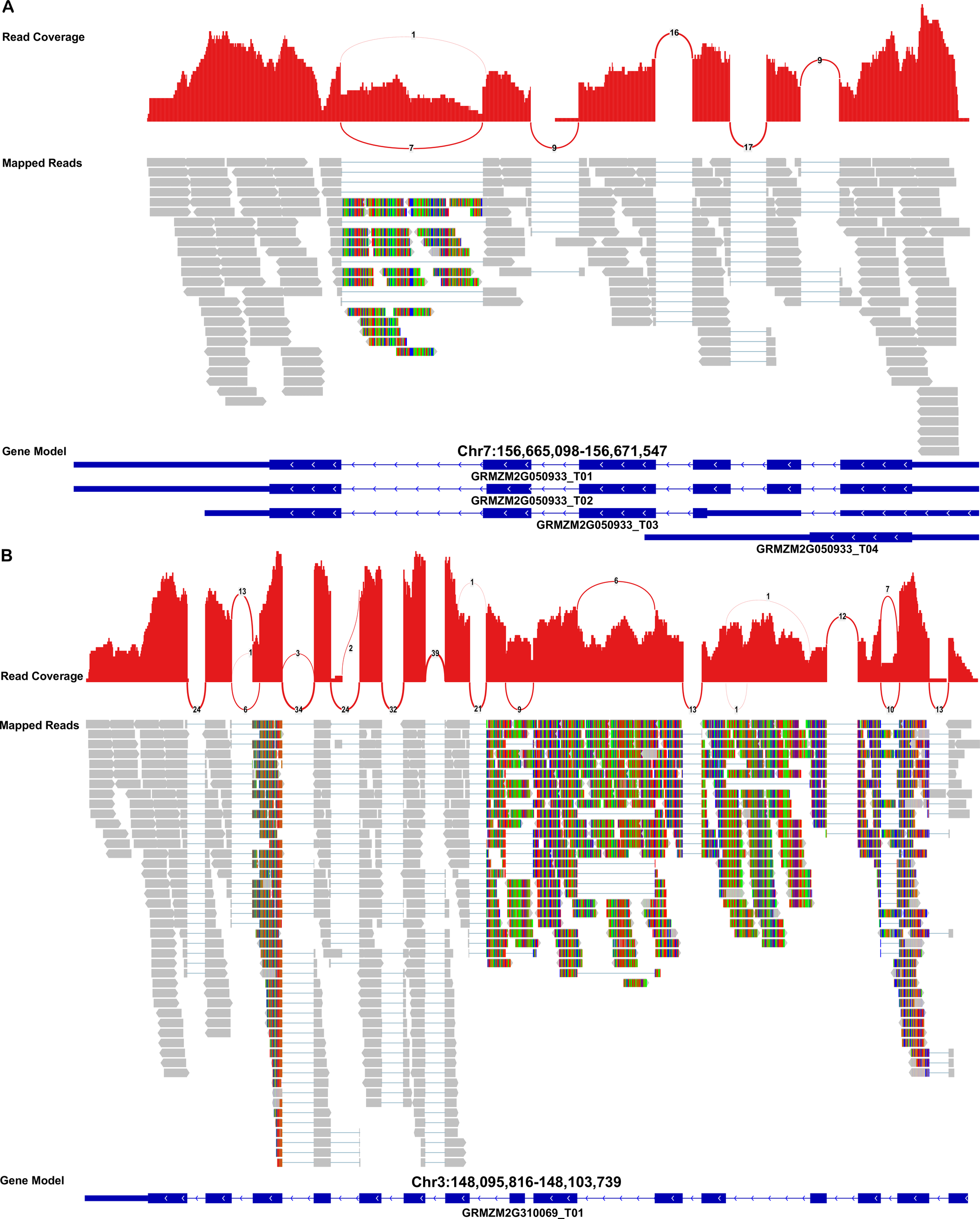

Supplement: S3 Fig — A, an example illustrating reads mapped to the gene (GRMZM2G050933) model, with a single AS event observed at 5 DAP. B, an example showing reads mapped to a gene (GRMZM2G310069) model, with multiple AS events observed at 10 DAP. The different colours represent different bases, and the coloured areas indicate the regions in which AS events occurred. Corresponding to Fig 2B and 2D. (TIF) [file pone.0163814.s003.tif]

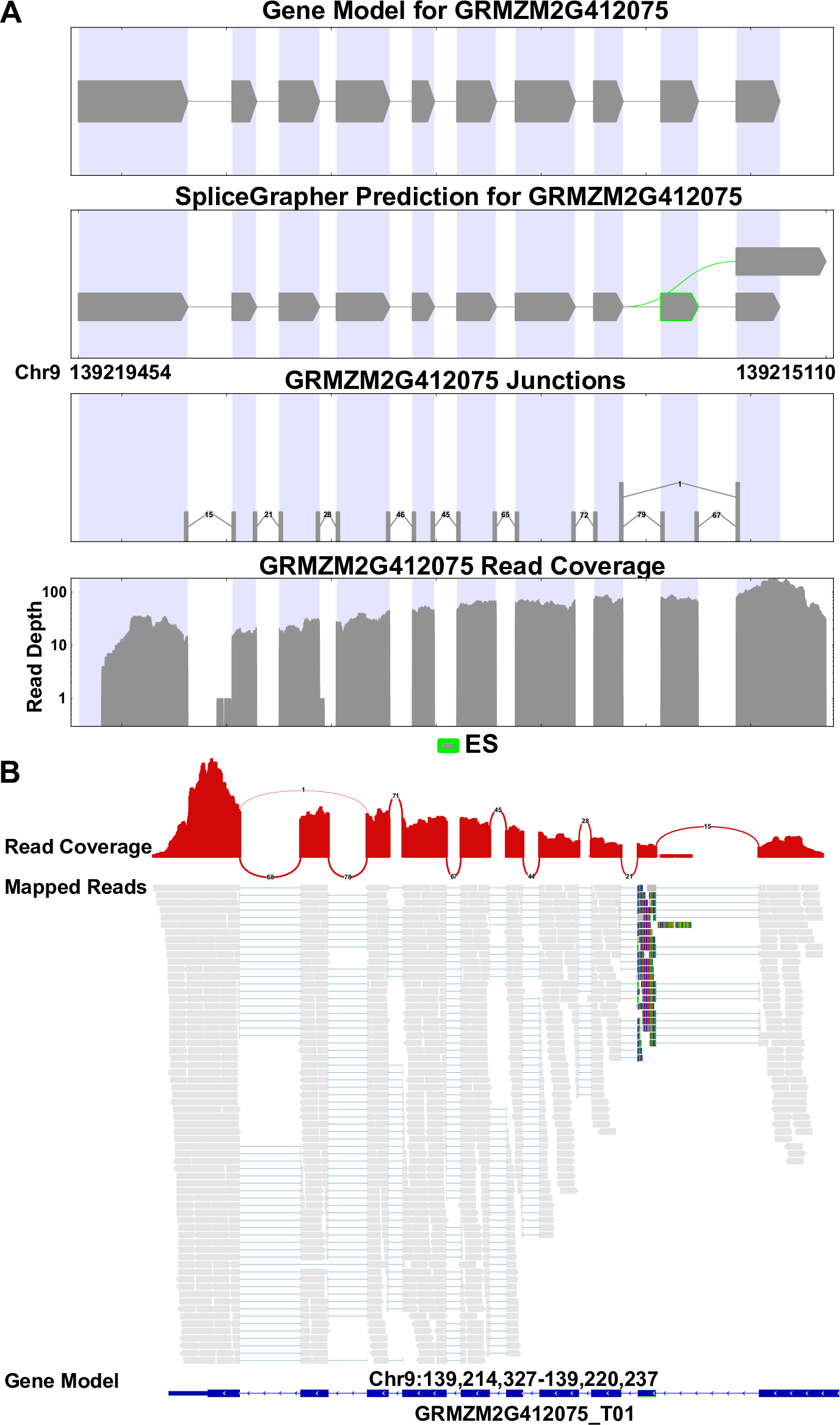

Supplement: S4 Fig — A and B, an example illustrating a newly identified transcribed regions, GRMZM2G412075, showing reads mapped to the gene model, with a single AS event observed at 15 DAP. The different colours represent different bases, and the coloured areas indicate the regions in which ES occurred. (TIF) [file pone.0163814.s004.tif]

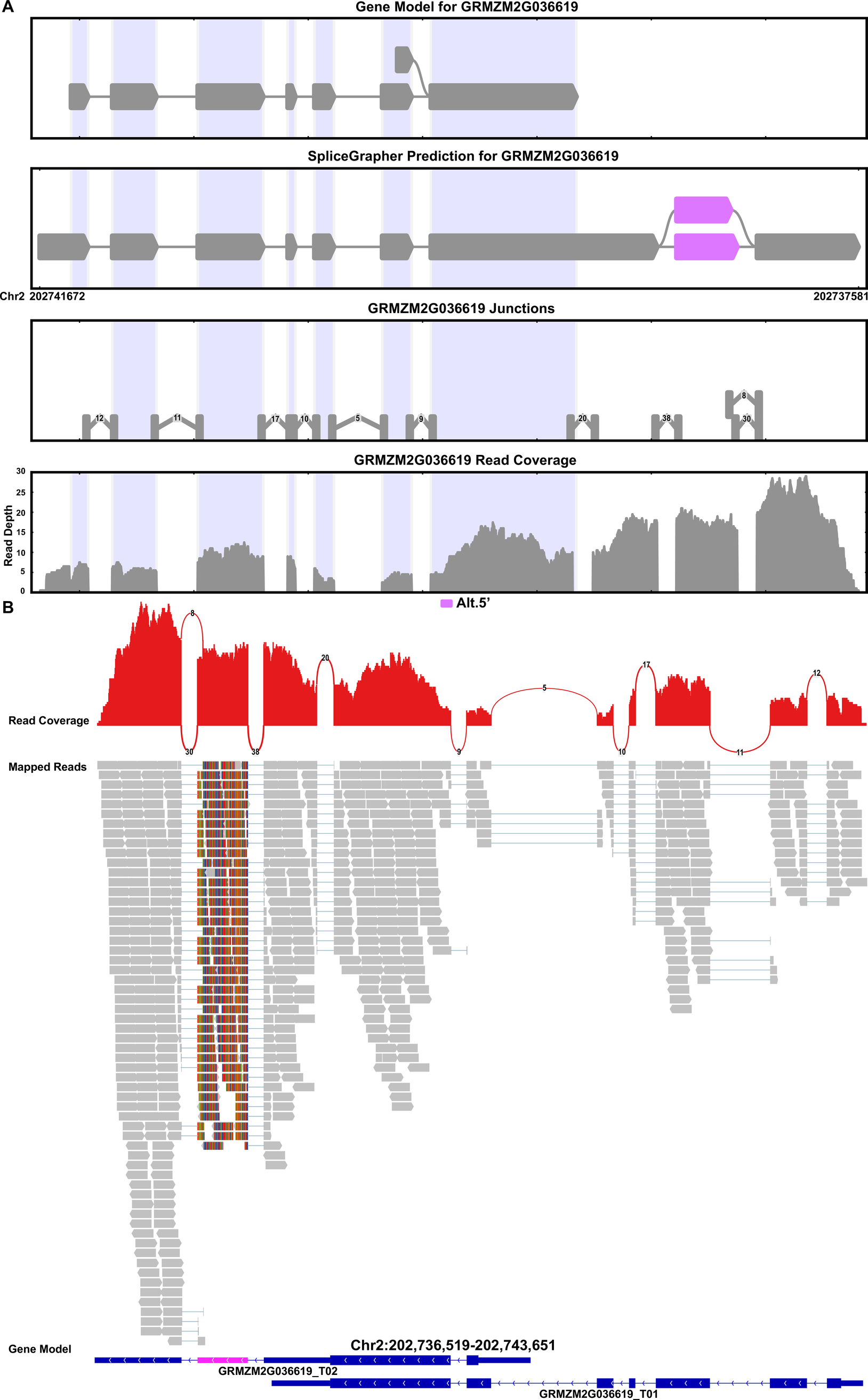

Supplement: S5 Fig — A and B, an example illustrating newly identified transcribed regions of GRMZM2G036619, showing reads mapped to the gene model, with a single AS event observed at 5 DAP. The different colours represent different bases, and the coloured areas are regions with an A5SS. (TIF) [file pone.0163814.s005.tif]

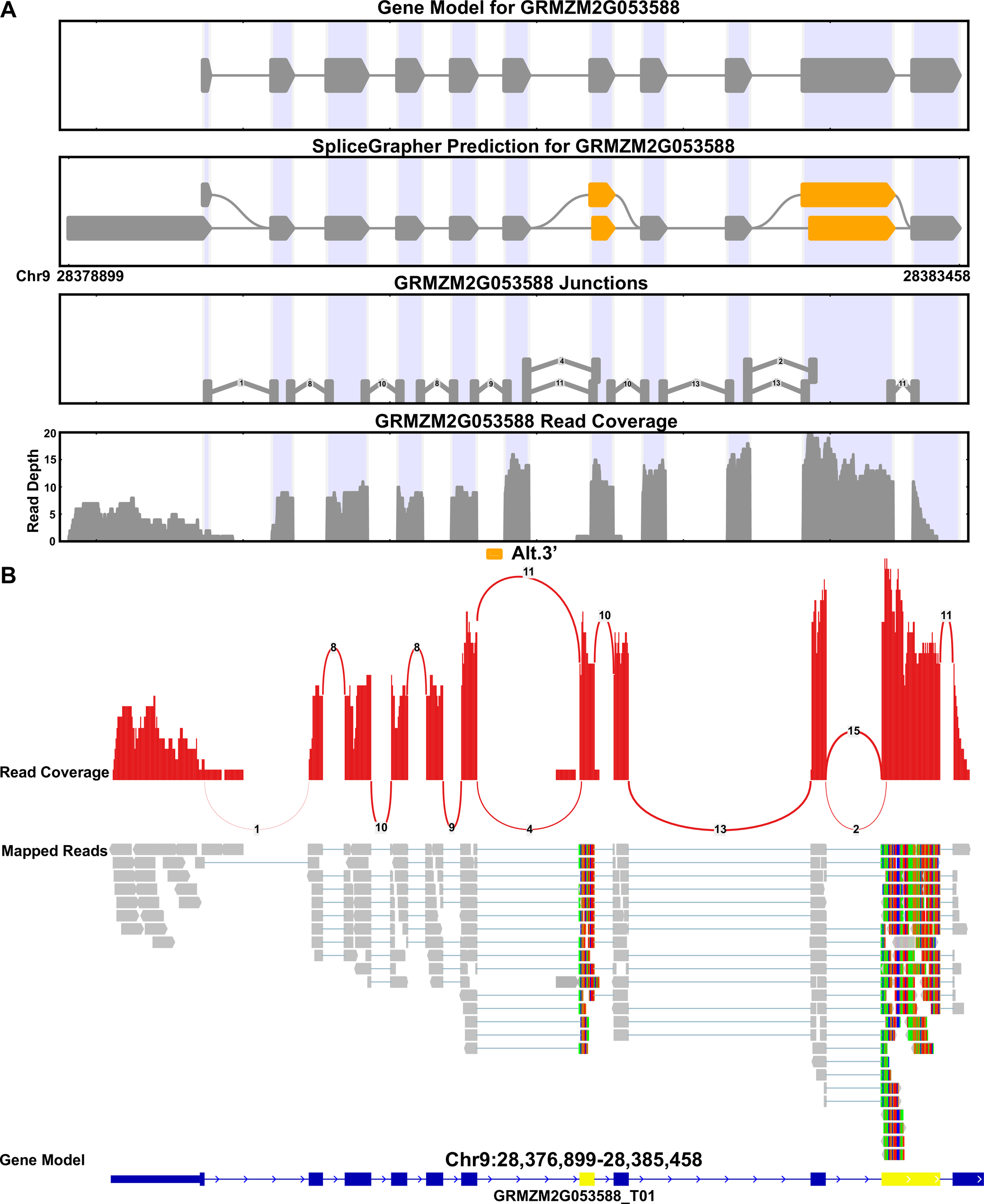

Supplement: S6 Fig — A and B, an example illustrating newly identified transcribed regions of GRMZM2G053588, showing reads mapped to the gene model, with a single AS event observed at 5 DAP. The different colours represent different bases, and the coloured areas are regions with an A3SS. (TIF) [file pone.0163814.s006.tif]

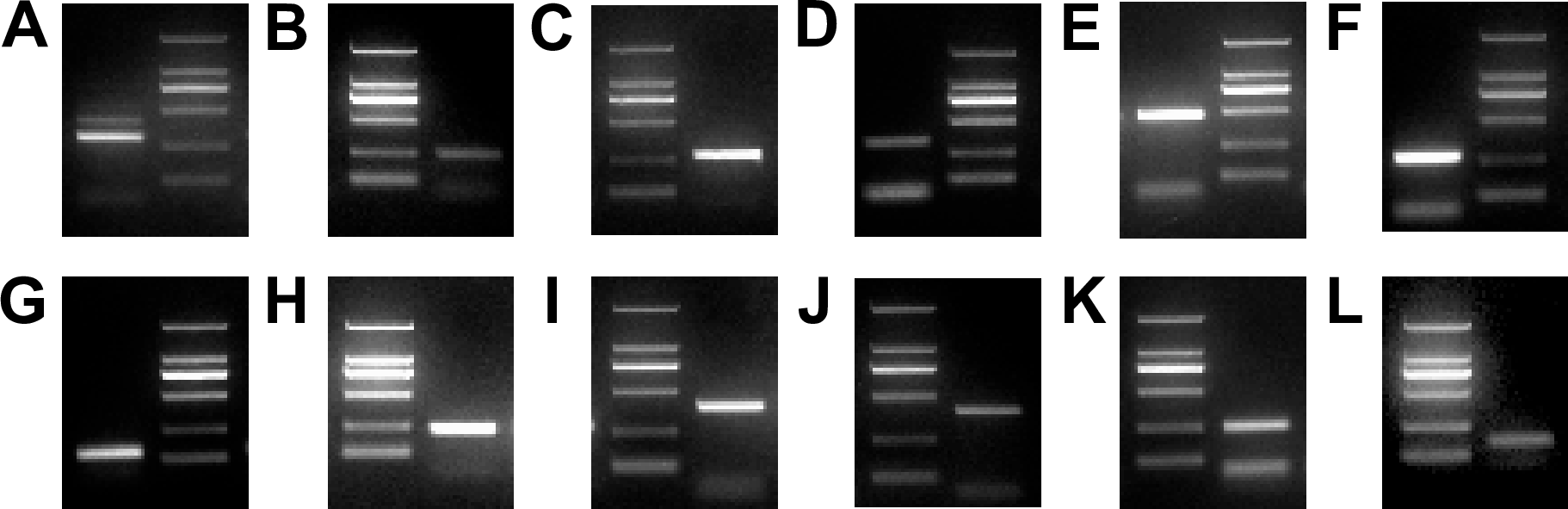

Supplement: S7 Fig — The capital letters indicate types of AS events. Corresponding to S1 Table. (TIF) [file pone.0163814.s007.tif]

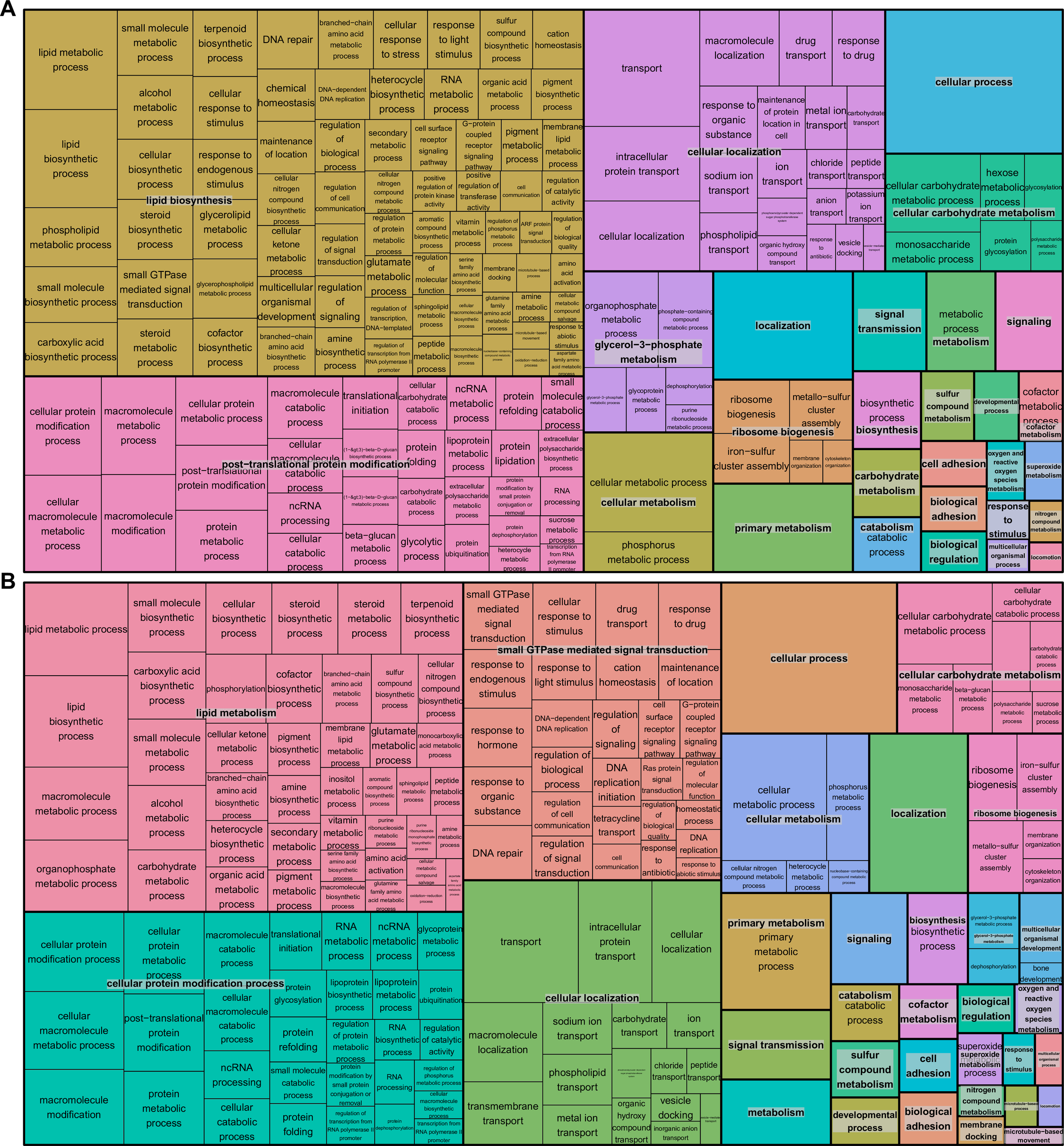

Supplement: S8 Fig — A, Enrichment of biological process-related GO terms of genes with a single AS event. B, Enrichment of biological process-related GO terms of genes with multiple AS events. (TIF) [file pone.0163814.s008.tif]

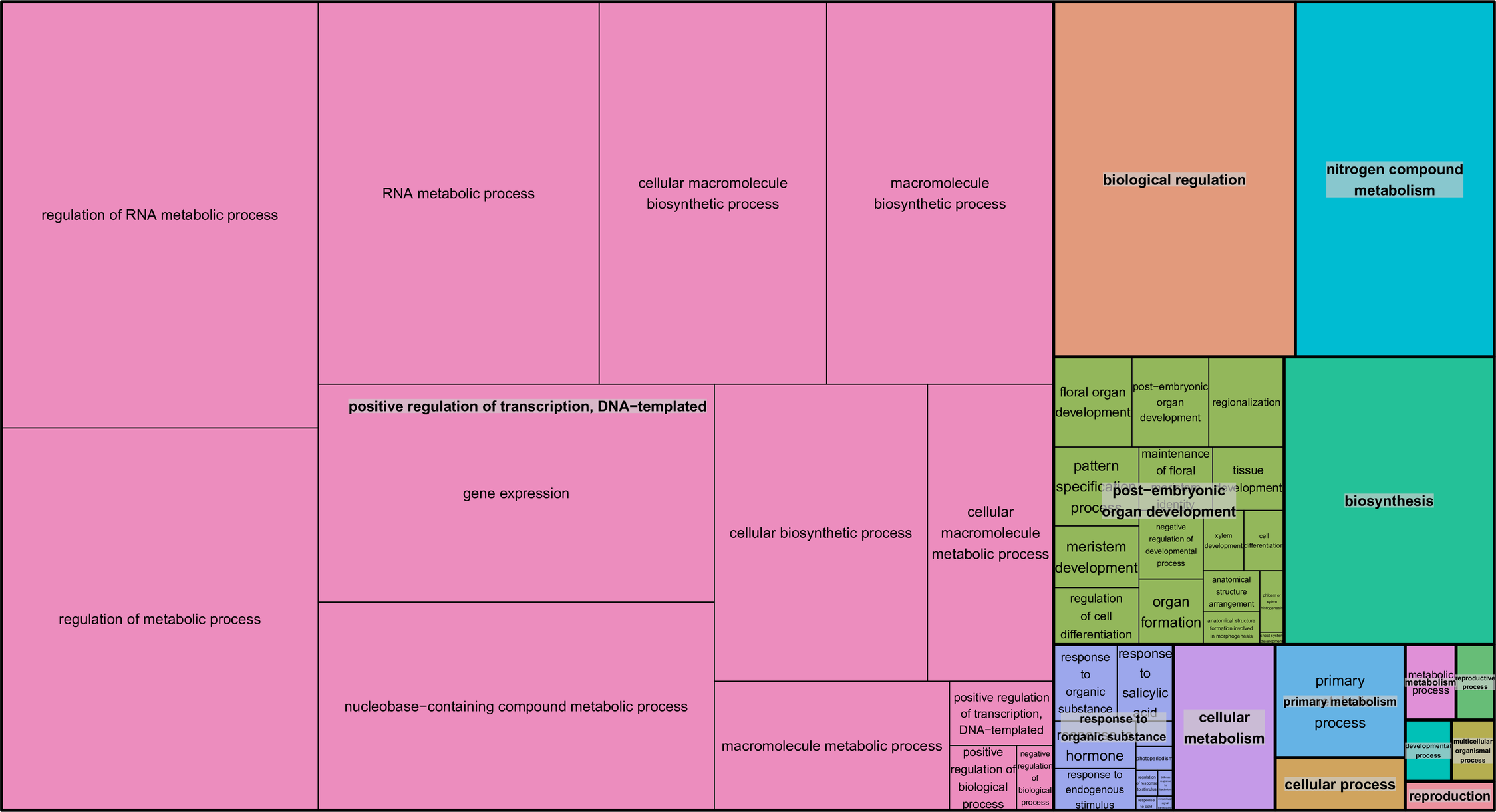

Supplement: S9 Fig — Enrichment of biological process-related GO terms of DETFs from four developmental stages of maize endosperm. (TIF) [file pone.0163814.s009.tif]
